# A study about the impact of indoor air pollution on cognitive function among middle-aged and older adult people in India

**DOI:** 10.1186/s13690-024-01286-5

**Published:** 2024-04-25

**Authors:** Subhadeep Saha, Priya Das, Tanu Das, Partha Das, Tamal Basu Roy

**Affiliations:** 1 Department of Geography, Raiganj University, Uttar Dinajpur, Raiganj, West Bengal 733134 India; 2 Department of Geography, University of Gour Banga, Malda, West Bengal 732101 India

**Keywords:** Cognitive decline, Housing environment, unhealthy lifestyle, Older adults, India

## Abstract

**Background:**

In the 21st century, people still use solid fuel for cooking at home, resulting in poor indoor air quality. Prolonged exposure to such conditions may negatively affect one’s cognitive function. So, the present study examines the possible association between IAP and the cognitive function of individuals aged 45 years or above in India.

**Methods:**

The study utilizes secondary data, procured from the longitudinal ageing study in India (2017-18). Treatment effects through regression-adjusted models were applied to represent the association between IAP and cognitive health and the results are represented by beta coefficient (β). Three separate models with a 95% confidence interval adjusting with the other factors like housing environment, individual and behavioural were framed.

**Results:**

The study revealed that households without a separate kitchen (β = -0.64; 95%CI: -0.90 to -0.39) and electricity (β = -0.97; 95%CI: -1.34 to -0.61) significantly affect cognitive strength. Cognitive decline is likely more pronounced among older adults (β = -1.19; 95%CI: -1.42 to -0.96) than the middle-aged population. Moreover, the cognitive ability of rural residents (β = -1.11; 95%CI: -1.49 to -0.73) and women (β = -2.05; 95%CI: -2.29 to -1.81) is negatively associated with IAP exposure. Older adults with no reading habits (β = -6.28; 95%CI: -6.72; to -5.85) and physical inactivity (β = -0.96; 95%CI: -1.22 to -0.70) had a sign of notable decline in cognitive ability.

**Conclusions:**

Findings revealed that cognitive function is negatively associated with IAP, demanding a deep intervention plan to minimize the detrimental effect.

**Table:** 

**Text box 1. Contributions to the literature**
• This study considers indoor air pollution (IAP) as a treatment effect on the cognitive decline of the individual which means measuring the cognitive decline (revealed through negative β co-efficient) separately adjusted with the factors like housing environment, background, and behavioural factors if respondents currently fall in IAP condition at the same time.
• The current study has considered middle-aged as well as older adults also to assess cognitive decline.
• Through this paper, people may feel the necessity of clean fuel use like LPG, electrical strove, etc. for cooking.

## Background

Almost all countries have been abiding by longer life expectancy [1]. The Census of India delineates that the proportion of the projected elderly population will be hiked by 19.5% by 2050 [2]. As people grow older, an eminent prospect of ageing biology modulates the length and standard of life [3]. On this wise, cognitive function is also linked to the well-being of older adults. Cognitive function is a mental aptitude that is required for our daily standard life activities. It comprises orientation, memory, arithmetic skills, object identification, and executive tasks [4]. However, there is an inverse relationship between an individual’s mental abilities and age. About 7.4% of older adults appeared to be suffering from dementia in India [5]. Globally, non-communicable diseases (NCDs) like dementia ranked in seventh position in the WHO’s top ten causes of mortality [6]. Evidence highlights that caring for Alzheimer’s disease or another dementia may overtake other NCDs [7]. Cognitive dysfunction can have crucial economic and social implications for older individuals, resulting in a lower quality of life [8, 9]. Several other factors associated with ageing place older adults at risk of cognitive impairment [10]. To this extent, using solid or unclean fuels within a household creates indoor air pollution (IAP) and initiates the circumstances of several health anomalies.

There are nine out of ten individuals breathe impure air globally [11]. Most air pollution is produced by human-induced day-to-day activities [12, 13] and it has affected the physical as well as mental health of individuals [14, 15]. Globally 6.7 million deaths occurred due to air pollution whereas household air pollution took nearly 3.2 million deaths of individuals in 2020 [16]. Using solid fuel inside the house may also form IAP, and cognitive performance remains a critical public health concern in developing countries like India. NHFS-IV reported that nearly 56.2% of households do not use clean fuel, leading to form IAP [17]. Burning solid fuels (dung, crop residue, and wood) without a ventilated cooking space generates numerous poisonous gases and particulate matter that might shape impaired mental ability. This situation mostly hits older adults, who are more likely to spend much of their time at home [18]. Several population studies have uncovered the fact that IAP alters healthy cardio-pulmonary and cognitive function [19–21].

Yet in developing countries like India, there are a limited number of studies on cognitive decline among older adults. Numerous empirical studies outlined the several risk factors that are associated with cognitive impairment but housing environmental-related risk factors remain in narrow attention relatively. Very few studies have highlighted the relationship between cognitive function and surrounding air pollution. Most of them focused only on women and children with relatively smaller sample sizes [14, 22]. However, surrounding air pollution affects the cognitive ability of individuals of all ages [23, 24]. So, this study has given its attention to understanding the impact of IAP at the household level and the cognitive ability of middle-aged and older adults in India. In this context, the following hypothesizes have been formulated:

i) Households deprived of basic amenities (without a separate kitchen, electricity, or proper sanitation and made up of temporary materials) are positively associated with a cognitive decline under the treatment effect of IAP.
ii) There is a significant relationship between cognitive dysfunction and older individuals’ background characteristics in the presence of IAP within the household.
iii) Individual Behavioural characteristics are significantly related to the cognitive impairments in the presence of treatment effect of IAP.

## Outcome variable

To assess cognitive function, the data released by LASI, followed the five different domains on assessment of mental ability based on the assignment of a specific range of scores among middle-aged and older adults.

- Memory: It consists of immediate word recall (score 0–10) and delayed word recall (score 0–10).
- Orientation: It consists of time (score 0–4) and place (score 0–4).
- Arithmetic function: It deals with backward counting (score 0–2), serial 7 (score 0–5), and computation (score 0–2).
- Executive function: It examines the ability of paper folding (score 0–3), and pentagon drawing (score 0–1).
- Object naming: Object naming score ranges from 0 to 2.

A composite cognitive index was measured by merging all the mentioned domains and a score ranging from 0 to 43 was developed. A higher cognitive score denotes higher cognitive function and vice versa. However, detailed explanations and assessments are mentioned in the LASI full report.v

### Treatment factors

Treatment factors execute their distinct impact on the differences in the mean outcome of cognitive ability owing to its presence or absence. Here, if the household was using solid or unclean fuel (kerosene, woods/shrubs, crop residue, coal, and dung cake) for cooking, boiling water, lighting, or any other purposes inside the room environment using traditional chulha (a type of stove made with bricks or earthen materials and run by solid or unclean fuel) or open fire without any ventilation were considered as having the signs of prevailing indoor air pollution (IAP).

## Covariates

Here the attached covariates are distinguished into three factors i.e., housing environment; individual and behavioural factors.

### Housing environment

Separate kitchen: Respondents were asked whether they had a separate kitchen and responses were recoded as “yes” and “No”.

Housing type: The present study classified the housing type into three categories i.e., houses made with temporary materials like mud, straw, bamboo, grass, and leaves, etc. coded as “Kutcha”, and houses made with any permanent materials like iron rods, brick, and cement, etc. coded as “Pucca”, and house made with the combination of both temporary and permanent materials coded as “Semi-pucca”.

Sanitation: at the time of the survey, Respondents were asked whether they had sanitation facilities. According to their responses present study categorized sanitation as “Yes” if respondents used flush toilets, pit latrines, twin pits, or composite toilets, and if they had no facility or used open space for sanitation categorized as “No”.

Electricity: During the survey, Respondents were asked whether they had an electric supply and responses were recoded as “yes” and “No”.

### Individual factors

Age: It was recoded as ‘45–59 years’ (middle-aged), ‘60–74 years’ (older adults), and ‘74 + years’ (elderly).

Sex: It was categorized as “Male” and “female”.

Residence: It was categorized as ‘and “Rural” and “urban”.

Attend school: If the respondents ever attended school coded as “Yes” whereas did not ever attend school coded as “No”.

Currently working: at the time of the survey if individuals engage in any kind of economic work coded as “Yes” and otherwise “No”.

Marital status: The present study classified the marital status into two groups “Currently in union” which means married and “Currently not in union” (if respondents were unmarried, deserted, separated, widowed, and divorced).

Sleeping problem: Respondents were inquired whether they experience sleep problems in days per week. Their responses were recoded as “yes” and “No”.

### Behavioural factors

Physical exercise: respondents were asked whether they do physical exercise like gardening, washing clothes, walking at a moderate pace, floor or stretching exercises, and fetching water. The present study classified their answer into three categories i.e., “Daily”, “Sometimes” and “Never”.

Reading habit: Individuals were questioned, ‘Do you read newspapers, magazines, and books?’ Their responses were classified their answer into three categories i.e., “Daily”, “Sometimes” and “Never”.

Tobacco consumption: If the respondents ever consumed any smoked or smokeless tobacco (gutka, pan masala, cigarettes, bidi, cheroot, hookah), it was recoded as “Yes” whereas they did not ever consume tobacco recoded as “No”.

Eat sufficiently: whether individual eats sufficiently or not based on four responses (i). ever reduce or skip meals (ii). Eat enough food (iii). Hungry but did not eat and (iv). Did not eat for a whole day. Their answer was recoded as “yes” and “No”.

Self-rated health (SRH): According to the rating of the respondents’ own responses this study was classified into three categories: “Good”, “Fair” and “Poor”.

## Study design and study population

The contemporary research utilizes a cross-sectional analysis by using the LASI wave-1 (The Longitudinal Ageing Study in India) dataset. LASI is the longitudinal survey that gathered information about 72,250 older adults aged 45 years or above, together with spouses. The wave-1 survey was organized from April 2017 to December 2018 and covered all Indian states and union territories. The survey gathered information about health, wealth financial resources, and social considerations. The survey made use of a multistage stratified area probability cluster sampling approach. The stage sampling design was operated in rural regions whereas the stage sampling design was applied in urban regions. The current study has identified respondents who are passing through 45 years of age and above. The total sample of 35,059 was screened (22,735 male and 12,324 female) middle-aged and older individuals after erasing the missing value.

### Analytical approach

The study applied the treatment effect through a regression adjustment model to explore the link between IAP and cognitive aptitude among India’s middle-aged and older adult population. Housing environmental characteristics are shown in Model 1. Individual characteristics and behavioural characteristics were represented in models 2 and 3 respectively. Housing environmental characteristics such as separate kitchens, housing type, sanitation, and electricity were regarded to be conformed in model (1) Individual characteristics such as age, sex, residence, working status school attendance, sleeping problems, and marital status were included to be fitted in model (2) Behavioural characteristics such as practicing physical exercise, having reading habits, tobacco consumption habits, food sufficiency, and SRH were regarded to be adjusted in model (3) The regression coefficient was estimated at a 95% confidence interval (CI). Under the presence of IAP, mean cognitive scores for each domain were also computed and compared subsequently. In addition, to compare with individual effect size two-way ANOVA along with effect size (eta-squared) was meant to measure for interaction effect and its effect strength between IAP and different domains. All the statistical analyses were done by STATA 17.

## Results

**Fig. 1:**
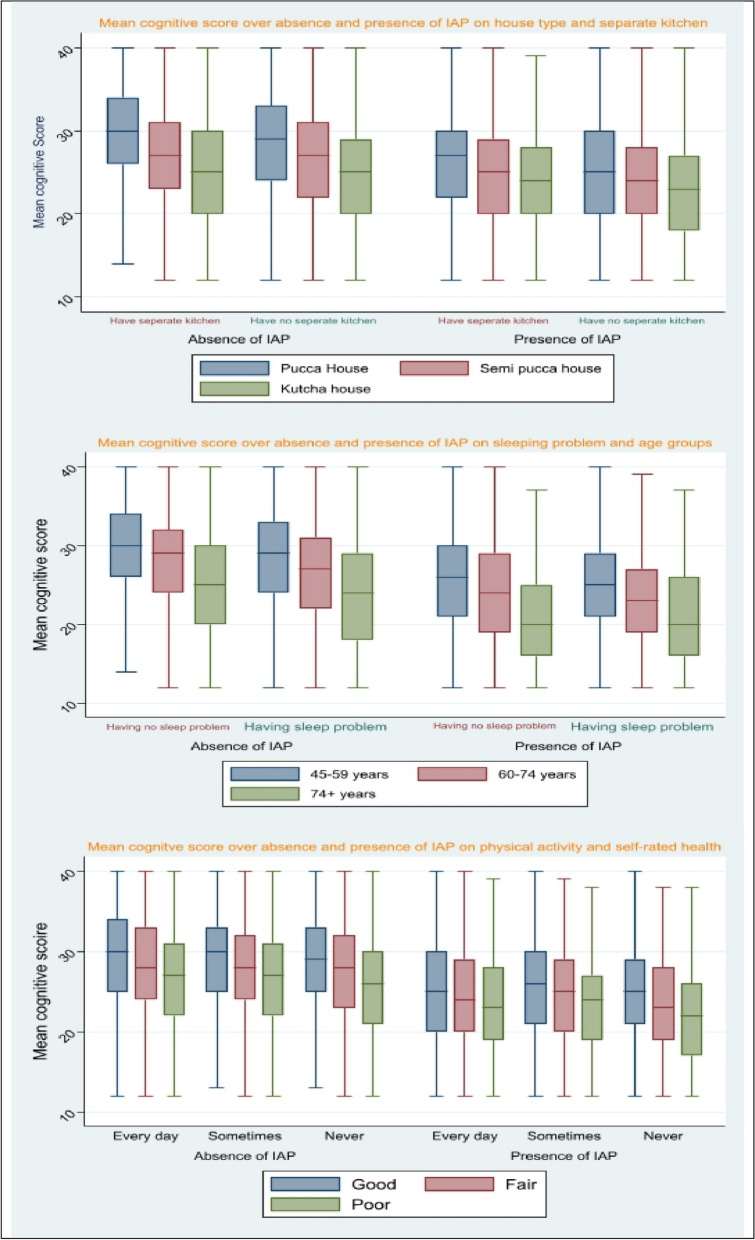
Visualize the cognitive decline in the presence and absence of IAP

Table 1 highlights the cognitive score of the participants along with the effect size between IAP and different housing environments, and individual and behavioural characteristics. In the case of the group variability test of the mean cognitive score of each household, individual and behavioural factor except reading habit, the interaction effect with IAP is wider than the individual effect. Respondents with no separate kitchen for cooking had significantly lower cognitive mean scores than those individuals who had a separate kitchen in their household for cooking (25.42 vs. 27.66). Participants living in kutcha houses, without sanitation facilities and electricity, reported lower cognitive scores than those older adults who used to live in pucca houses (23.97 vs. 28.54), had sanitation (27.63 vs. 23.92) and electricity facilities (27.18 vs. 23.15). Boxplot visualizes how mean cognitive score differs in the absence and presence of IAP in the various house types if the individual had no separate kitchen (Fig. 1).

**Table 1:** Mean cognitive score of the study population and individual and interaction effect between indoor air pollution and different household, individual and behavioural characteristics

**Variable**	**Sample**	**Percentage**	**Mean cognitive score (SE)**	**95% CI**		**Eta-squared**		
						*Individual effect size*	*Interaction effect*	
							*Effect size*	*p-value*
**Separate kitchen**								
Yes	24063	68.64	27.66 (0.04)	27.58	27.74	0.009	0.050	*
No	10996	31.36	25.42 (0.06)	25.30	25.54			
**Type of house**								
Pucca	18883	53.87	28.54 (0.04)	28.45	28.63	0.031	0.033	*
Semi-pucca	9845	28.10	25.84 (0.06)	25.72	25.96			
Kutcha	6331	18.03	23.97 (0.08)	23.82	24.12			
**Sanitation**								
Yes	28698	81.86	27.63 (0.03)	27.56	27.70	0.024	0.045	*
No	6361	18.14	23.92 (0.08)	23.77	24.08			
**Electricity**								
Yes	33108	94.43	27.18 (0.03)	27.11	27.25	0.010	0.055	*
No	1951	5.57	23.15 (0.14)	22.87	23.42			
**Age in years**								
45–59	19360	55.22	28.04 (0.04)	27.96	28.13	0.039	0.065	*
60–74	12849	36.65	26.12 (0.06)	26.01	26.23			
74 +	2850	8.13	23.38 (0.13)	23.13	23.63			
**Sex**								
Male	22735	64.85	28.35 (0.04)	28.28	28.43	0.077	0.063	*
Female	12324	35.15	24.38 (0.06)	24.27	24.50			
**Residence**								
Urban	12184	34.75	29.49 (0.05)	29.39	29.59	0.013	0.031	*
Rural	22875	65.25	25.61 (0.04)	25.53	25.69			
**Ever attend school**								
Yes	21296	60.74	29.88 (0.04)	29.82	29.95	0.245	0.024	*
No	13763	39.26	22.43 (0.05)	22.34	22.52			
**Currently working**								
Yes	23119	65.94	27.58 (0.04)	27.50	27.66	0.020	0.071	*
No	11940	34.06	25.76 (0.06)	25.64	25.88			
**Marital status**								
Currently in union	28057	80.02	27.62 (0.04)	27.55	27.70	0.038	0.066	*
Currently Not in Union	7002	19.98	24.28 (0.08)	24.12	24.44			
**Sleeping problem**								
No	21944	62.60	27.51 (0.04)	27.43	27.60	0.009	0.066	*
Yes	13115	37.80	26.02 (0.06)	25.92	26.13			
**Physical exercise**								
Every day	18891	53.88	27.14 (0.05)	27.05	27.24	0.003	0.067	*
Sometimes	5304	15.12	27.21 (0.08)	27.04	27.38			
Hardly ever or never	10864	31.00	26.51 (0.06)	26.39	26.63			
**Reading habit**								
Daily	6568	18.73	31.85 (0.06)	31.74	31.96	0.117	0.026	*
Sometimes	6597	18.83	29.74 (0.06)	29.62	29.86			
Never	21894	62.44	24.65 (0.04)	24.57	24.73			
**Tobacco consumption**								
No	19800	56.48	27.18 (0.04)	27.08	27.27	0.000	0.068	*
Yes	15259	43.52	26.67 (0.05)	26.58	26.77			
**Eat sufficiently**								
Yes	19884	56.71	27.32 (0.04)	27.23	27.41	0.004	0.066	*
No	15175	43.29	26.49 (0.05)	26.38	26.59			
**Self-rated health**								
Good	15751	44.93	27.92 (0.05)	27.82	28.02	0.017	0.064	*
Fair	14270	40.70	26.54 (0.05)	26.43	26.64			
Poor	5038	14.37	25.14 (0.09)	24.97	25.32			

Eta-squared was obtained through one way & two-way ANOVA

Individuals belonging to the age group, i.e., 74 years and above had the lowest score than those belonging to the age group 45 years and above (23.38 vs. 28.04). Older women had notably lower cognitive scores than the older men (24.38 vs. 28.35). Participants living in a rural region, who never attended school, did not engage in work, were currently not in a union, and suffered from sleeping problems revealed lower mean cognitive scores than those participants who lived in an urban region (29.49 vs. 25.61), attended school (29.88 vs. 22.43), having engagement in work culture (27.58 vs. 25.76), currently in union (27.62 vs. 24.28) and did not suffer from sleeping problem (27.62 vs. 26.02) respectively. Boxplot displays how much cognitive scores vary in the absence and presence of IAP in the different age groups if the individuals suffer from sleeping problems (Fig. 1).

Middle-aged and older adults who did not have any reading habits reported lower cognitive scores than those individuals who had regular reading habits (24.65 vs. 31.85). Respondents who did not perform physical exercise regularly, consumed smoke/smokeless tobacco, could not eat sufficiently, and self-reported as having poor health affirmed lower cognitive scores than their counterparts. Boxplots represent how much cognitive score changes in the absence and presence of IAP in different self-rated health if the individuals did not do physical exercise regularly (Fig. 1).

Table 2 highlights the estimation of the treatment effect of IAP on the overall cognitive score through regression adjusting for housing environmental, individual, and behavioural characteristics. A beta coefficient with 95% CI was calculated to understand the association and significant level. Looking at the housing environmental characteristics, results showed that middle-aged as well as older adults affected by IAP and having no separate kitchen (β = -0.64; 95% CI = [-0.90 to -0.39]), sanitation (β = -0.81; 95% CI = [-1.07 to -0.55]), and electricity (β = -0.97; 95% CI = [-1.34 to -0.61]) were negatively associated with mean cognitive scores compared to those who had separate kitchen, sanitation, and electricity. In addition, respondents who were in IAP and used to live in semi-pucca (β = -0.87; 95% CI = [-1.18 to -0.57]) and kutcha (β = -1.79; 95% CI = [-2.10 to -1.48]) houses were negatively associated with average cognitive scores than those who used to live in pucca houses.

**Table 2:** Estimation of treatment effects of indoor air pollution on the overall cognitive score through regression adjusting for housing, individual and behavioural characteristics

	**Model-1 adjusted beta coefficient (95% CI)**	**Model-2 adjusted beta coefficient (95% CI)**	**Model-3 adjusted beta coefficient (95% CI)**
**Variables**	***Presence of IAP***		
	***Housing characteristics***	***Individual characteristics***	***Behavioural characteristics***
**Separate kitchen**			
Yes	**Ref**		
No	-0.64***(-0.90 to -0.39)		
**Type of house**			
Pucca	**Ref**		
Semi-pucca	-0.87***(-1.18 to -0.57)		
Kutcha	-1.79***(-2.10 to -1.48)		
**Sanitation**			
Yes	**Ref**		
No	-0.81***(-1.07 to -0.55)		
**Electricity**			
Yes	**Ref**		
No	-0.97***(-1.34 to -0.61)		
**Age in years**			
45-59		**Ref**	
60-74		-1.19***(-1.42 to -0.96)	
74 +		-3.17***(-3.59 to -2.74)	
**Sex**			
Male		**Ref**	
Female		-2.05***(-2.29 to -1.81)	
**Residence**			
Urban		**Ref**	
Rural		-1.11***(-1.49 to -0.73)	
**Ever attend school**			
Yes		**Ref**	
No		-4.93***(-5.17 to -4.70)	
**Currently working**			
Yes		**Ref**	
No		-0.51***(-0.76 to -0.26)	
**Marital status**			
Currently in union		**Ref**	
Currently Not in Union		-0.93***(-1.21 to -0.66)	
**Sleeping problem**			
No		**Ref**	
Yes		-0.27***(-0.48 to -0.06)	
**Physical exercise**			
Every day			**Ref**
Sometimes			0.25 (-07 to 0.58)
Hardly ever or never			-0.96***(-1.22 to -0.70)
**Reading habit**			
Daily			**Ref**
Sometimes			-1.21***(-1.70 to -0.72)
Never			-6.28***(-6.72 to -5.85)
**Tobacco consumption**			
No			**Ref**
Yes			0.88 (0.66 to 1.10)
**Eat sufficiently**			
Yes			**Ref**
No			-0.61***(-0.84 to -0.39)
**Self-rated health**			
Good			**Ref**
Fair			-0.97***(-1.21 to -0.72)
Poor			-1.98***(-2.31 to -1.64)
**Constant**	26.07 (25.83-26.32)	30.24 (29.86-30.63)	30.32 (29.85-30.80)
**POmeans**			
*Absence of IAP*	27.35 (27.27-27.43)	27.30 (27.22-27.38)	27.55 (27.47-27.62)
*Presence of IAP*	25.10 (24.95-25.25)	25.71 (25.56-25.86)	25.35 (25.22-25.48)

Older adults aged between 60 and 74 years and proximate to IAP had a negative association in terms of cognitive score than middle-aged people (β = -1.19; 95% CI = [-1.42 to -0.96]). In addition, if any older women (β = -2.05; 95% CI = [-2.29 to -1.81]), rural residents (β = -1.11; 95% CI = [-1.49 to -0.73]), were prone to IAP were adversely correlated with the mean cognitive score than older men and urban residents respectively. Individuals who never attended school (β = -4.93; 95% CI = [-5.17 to -4.70]), and at present not engaged in any kind of work (β = -0.51; 95% CI = [-0.76 to -0.26]), and are currently not in union (β = -0.93; 95% CI = [-1.21 to -0.66]) were unfavourably interrelated with a mean cognitive score if they were exposed to IAP.

Respondents who were exposed to IAP as well as didn’t have the habit of physical exercise regularly (β = -0.96; 95% CI = [-1.22 to -0.70]), and had no reading habit (β = -6.28; 95% CI = [-6.72; to -5.85]) were negatively associated with cognitive scores than those who did physical exercise and had reading habits regularly. Individuals who did not eat sufficiently (β = -0.61; 95% CI = [-0.84; to -0.39]), and reported their health as poor (β = -1.98; 95% CI = [-2.31 to -1.64]) were negatively correlated with average cognitive scores if they were exposed to IAP compared to those who could eat sufficiently and reported their health as good.

## Discussion

The presence of IAP and its detrimental effect on cognitive function among older adults become a vital health concern. Several earlier studies have demonstrated its association with cognitive health, but it is still unexplored how much mental ability declined in respect of housing environment, and individual and behavioural characteristics in association with IAP. The study has made it possible to unearth the issue among middle-aged and older adult people in the Indian context. Results from our study identified that IAP owing to the use of unclean fuel contributes to cognitive dysfunction, suggesting the use of cleaner fuel in households.

However, the present study is incapable of exploring the possible mechanism by which IAP can impact cognitive dysfunction among individuals. A chain of earlier works of literature has found that due to outdoor air pollution, contains health-damaging pollutants like carbon monoxide, sulfur dioxide, nitrogen oxide, fine particulate matter, and formaldehyde which create indoor air pollution [25–27]. Hence it may be feasible that such pollution due to solid fuel use affects the brain and mental ability in a similar mechanism. Many studies have established that through the air-blood carrier of the lungs, ultrafine particles enter the systematic circulation of the body and may directly [28] or via olfactory nerve translocate brain tissue [29]. Moreover, such particles accelerate the oxidative stress of the brain and neuroinflammation [30, 31]. If the presence of particulate matter [PM 2.5] remains high in outdoor air, it lessens the cerebral volume and white matter concentration in the brain [32]. Following this statement, it can be imaginable that IAP may reduce cognitive ability by the same biological process; nevertheless, which pollutants affect mental health and their definite mechanism remain obscure.

Worldwide studies about the relationship between cognitive decline and individual factors have been well-established [33, 34]. In addition, the study explored the association between cognition and individual characteristics in India if individuals were exposed to IAP. IAP had a more notably detrimental effect on older adults’ cognitive functioning than middle-aged people; the current study highlighted this effect. Moreover, our study has mentioned that cognitive ability was negatively associated with rural residents. It is often seen that rural households rely on solid fuel for cooking [35] which is the major contributing factor of IAP. Perhaps, the worst condition hit the females who were likely to spend more time on cooking purposes and reported lower cognitive scores [36]. Our study also noticed that individuals who were not involved in any work expressed negative cognitive scores. It may be possible as such individuals spend their significant time at home  [37]. In our study, middle-aged and older adults who were exposed to IAP and currently not in union revealed negative cognitive function. Depression for widowhood plays an important role in cognitive impairment [38]. In addition, several studies showed that widows were more possibly to stay at home and likely to spend time with their family members [39]. The long time spent at home may be a chance for exposure to IAP. Poor sleep and IAP have a deleterious effect on cognitive health as these factors generate oxidative stress in the brain [30, 40]. In affirmed with this statement current study found that middle-aged and older adults who had sleeping problems reported cognitive decline due to IAP exposure.

In this study, cognitive dysfunction was observed who had no separate kitchen for cooking, pucca house for living, sanitation, and electricity. Globally various environmental studies explained that IAP is increased owing to cooking without a separate kitchen as it lessens the indoor air quality by producing particulate matter and harmful gases [41–43]. People who had no electric facilities could not access electric stoves, microwave ovens for cooking, and exhaust fans to remove the gasses from indoors [44, 45] and they were more likely to depend on using unclean fuel for cooking purposes without ventilation which ultimately led to increased IAP. This condition is mostly prevalent in rural areas [46]. It is noticed that semi-pucca or kutcha houses are made without a ventilation system and may be clogged with smoke for a long time due to damp conditions [9, 47, 48]. This situation is possibly enough for the creation of IAP which hampers the mental well-being of individuals. Moreover, if older adults do not access proper sanitation probably report poor cognitive health [49].

Our study explored that certain behavioural characteristics such as not having any reading habits, fair and poor self-rated health, insufficient eating habits, and not exercising physical activity stood out as remarkable risk factors for cognitive ailment in the presence of IAP. Tobacco consumption may be directly associated with cognitive impairment [50] but our study found it insignificant. The possible reason may be that only smoked tobacco consumption may escalate IAP [51] and this study considers both smoked and smokeless tobacco. It is believed that physical activities boost mental health [52] but staying in IAP with physical inactiveness damages cognitive health [53]. Seeing this adverse effect on physical as well as mental health, foreign countries like Canada, Australia, the US, and Great Britain have suggested ‘Physical activity guidelines for older individuals [54]. Considering the economic, and social along with different cultural alignments within India, it is obligatory to undertake bold steps against IAP, thereby reducing its effect on cognitive dysfunction. Otherwise, the growing aged population may suffer from this health effect in the upcoming days.

### Advantages and shortcomings of the study

The study’s core advantage is that it utilized the most recent national representative elderly data from LASI from 2017 to 2018. The study assessed the association between cognitive ability and each variable as mentioned earlier in the light of IAP. In addition, the present study examined the association between housing environment and cognitive ability, which the other studies mostly overlooked. Overall, we can say this study tries to give evidence of cognitive decline through IAP which is produced from the use of solid fuel. This evidence may surely bear a message to the public service intervention to mitigate the IAP. At the same time, people may feel the necessity of why ventilation systems and clean fuel use like Liquified Natural Gas (LPG) are required for cooking.

Regardless of all the mentioned advantages, our study bears some shortcomings too. Firstly, as the study is dependent on cross-sectional data, the causal association cannot be justified. Utilizing the longitudinal data from further round surveys controls this limitation and establishes the causal relationship. Secondly, which pollutants and their intensity form the IAP cannot be determined. Thirdly, a specific mechanism for how cognitive ability is declined by IAP cannot be discovered. There may be other confounding variables that were not considered in the study. Lastly, it is doubtful that the study result solely represents the IAP as there was no estimation of ambient air pollution or other chemical risk to which individuals are likely to be exposed.

## Conclusion

Despite having a few drawbacks, the study highlighted the shreds of evidence of cognitive decline among middle-aged and older adults under different conditions. The outcome of the study has significant implications for public health concerns. Increased attention on policy-making in India is necessary where a large proportion of households still use different types of solid fuel. However, the Pradhan Mantri Ujjwala Yojana scheme has been inaugurated to encourage the use of LPG for women. However, its impact on reducing indoor air pollution at the household level is unrecognizable. In addition, more research on the effect of solid fuel use on older adults’ physical and cognitive health and urgent public service interventions may help to reduce this complicated and glaring concern.

## Data Availability

No datasets were generated or analysed during the current study.

## Abbreviations

NCD: Non-communicable disease
IAP: Indoor air pollution
SRH: Self-rated health
CI: Confidence interval
